# Estimation of withdrawal interval recommendations following administration of fenbendazole medicated feed to ring-necked pheasants (*Phasianus colchicus*)

**DOI:** 10.3389/fvets.2024.1444009

**Published:** 2024-07-31

**Authors:** Marta Carreño Gútiez, Melissa A. Mercer, Beatriz Martínez-López, Ronald W. Griffith, Scott Wetzlich, Lisa A. Tell

**Affiliations:** ^1^Department of Medicine and Epidemiology, Center for Animal Disease Modeling and Surveillance (CADMS), School of Veterinary Medicine, University of California, Davis, Davis, CA, United States; ^2^Department of Veterinary Medicine and Epidemiology, School of Veterinary Medicine, University of California, Davis, Davis, CA, United States; ^3^Department of Veterinary Microbiology and Preventive Medicine, College of Veterinary Medicine, Iowa State University, Ames, IA, United States

**Keywords:** fenbendazole, withdrawal time, drug residue, EMA, FDA, pheasants, food safety, tissue residue depletion

## Abstract

**Introduction:**

Prescribing fenbendazole medicated feed for pheasants in the USA is considered extra-label drug use under CPG Sec 615.115, and a safe estimated withdrawal interval (WDI) must be applied following administration to this minor food-producing species. This study sought to determine the pharmacokinetic and residue depletion profile for fenbendazole and its major metabolites to estimate a WDI for pheasants following fenbendazole administration as an oral medicated feed.

**Method:**

Pheasants (*n* = 32) were administered fenbendazole as an oral medicated feed (100 ppm) for 7 days. Fenbendazole, fenbendazole sulfoxide, and fenbendazole sulfone (FBZ-SO_2_) in liver and muscle samples were analyzed using HPLC-UV. Tissue WDIs were estimated using FDA, European Medicines Agency (EMA), and half-life multiplication methods for US poultry tolerances, EMA maximum residue limits, and the analytical limit of detection (LOD; 0.004 ppm). Terminal tissue elimination half-lives (T_1/2_) were estimated by non-compartmental analysis using a naïve pooled data approach.

**Results:**

The tissue T_1/2_ was 14.4 h for liver, 13.2 h for thigh muscle, and 14.1 h for pectoral muscle. The maximum estimated withdrawal interval was 153 h (7 days) for FBZ-SO_2_ in pectoral muscle using the FDA tolerance method (95% confidence interval for the 99^th^ percentile of the population), and the LOD as the residue limit.

**Discussion:**

The results from this study support the use of FBZ-SO_2_ as the marker residue in the liver of pheasants and the provision of evidence based WDIs following the extra-label administration of fenbendazole medicated feed (100 ppm) for 7 days.

## 1 Introduction

Pheasants (*Phasianus colchicus*) are classified by the US Food and Drug Administration (FDA) Center for Veterinary Medicine (CVM) as a minor food-producing species in the US (1), with 7,790,734 pheasants sold live in 2017 for use in sport, meat, and export (2). There are only a few FDA approved medications for treating pheasants including bacitracin methylenedisalicylate, bacitracin zinc, and amprolium (3). Therefore, due to the lack of available FDA approved medications for this species, extra-label drug use (ELDU) is permitted under the Animal Medicinal Drug Use Clarification Act (4). This veterinary product is often necessary for medical treatment.

Fenbendazole is a benzimidazole-class anthelmintic that is FDA-approved for use in several food-animal species, including as an oral medicated water in chickens (broilers and layers) and as a medicated feed in growing turkeys in the USA. It has demonstrated efficacy against immature and adult stages of nematodes, tapeworms, and trematodes in domestic food-animal species (5). Ring-necked pheasants raised on propagation farms are commonly parasitized with the nematode *Syngamus trachea* (Gapeworm), which can result in significant flock morbidity and mortality (6, 7). Since fenbendazole has demonstrated efficacy against *Syngamus trachea* in a variety of avian species, including pheasants, fenbendazole oral medicated feed is an attractive therapeutic option for use in pheasants (8). Following consumption by turkeys and chickens, fenbendazole is rapidly metabolized to fenbendazole sulfoxide and fenbendazole sulfone (9). The marker residue in the US is fenbendazole sulfone in the liver for chickens and turkeys. However, the metabolism of fenbendazole and tissue distribution of metabolites in pheasants is incompletely understood, prohibiting the ability to determine the marker residue in pheasants. Additionally, tissue pharmacokinetic data is required to estimate withdrawal intervals (WDIs) following ELDU of fenbendazole in pheasants.

While AMDUCA prohibits the use of drugs in an extra label manner in feed (4), the FDA adopted a Compliance Policy Guidance stating that regulatory discretion would be applied to the prescription of medicated feed in an extra-label manner for treating minor food-animal species, such as pheasants, for up to 6 months (10). One of the greatest challenges for clinical veterinarians in the USA is that the veterinarian is responsible for providing a greatly extended withdrawal recommendation for food products following ELDU in food animals. The FDA-CVM approved withdrawal time (WDT) applied only to products administered according to label directions, and is defined as the interval of time that occurs between the last administration of a drug and the time when the animal can be safely slaughtered for human consumption (11). An animal tissue or product is determined to be deemed safe for human consumption when drug concentrations are below limits established by the FDA (defined as “tolerance”) or European Medicines Agency (EMA; defined as maximum residue limit [MRL]) for the target tissue and marker drug residue. For the FDA, the WDT calculated as the time when the upper one-sided 99% tolerance limit for the residue is below the tolerance with 95% confidence—meaning that the 99^th^ percentile of animals' tissue drug concentrations will fall under the tolerance with a 95% confidence interval (12). While both the FDA and EMA use 95% confidence intervals, the EMA differs by the use of a 95% upper one-sided tolerance limit, which reduces the weight of extreme extrapolation in these calculations (13).

Since veterinarians are directly responsible for providing a safe estimated WDI for ELDU in the US, more simplified approaches that minimize the need for extensive data or sophisticated software are often preferred. Estimated WDIs vary depending on the method used to calculate them, and the methodology used largely depends on the available pharmacokinetic data. When estimating a WDI, if no FDA tolerance exists for the drug in the treated species and food product of interest, then the analytical limit of detection (LOD) is typically used in place of the tolerance/MRL. Since >99% of a drug is depleted from a tissue after 10 elimination half-lives (t_1/2_), one of the most common methods of estimating a WDI following ELDU is to multiply the terminal tissue or product t_1/2_ by a factor of 10 (14). However, this half-life multiplication method does not account for population variability, and therefore it is more robust to apply regulatory tolerance methods to pharmacokinetic study data to estimate WDI following ELDU if the data is available.

The purpose of this study was to estimate WDI recommendations for pheasants following administration of fenbendazole medicated feed [100 parts-per-million (ppm)] for 7 days. The specific aims of this study were to: (1) characterize the depletion of fenbendazole and its metabolites in tissues following continuous feeding of oral medicated feed (100 μg/g or ppm) for 7 days and (2) compare the estimated WDIs for pheasants fed with fenbendazole medicated feed as calculated by the FDA tolerance, EMA MRL, and half-life multiplication methods.

## 2 Materials and methods

### 2.1 Pheasants

A group of healthy 40 ring-necked pheasants approximately 11 weeks of age, twenty of each sex, were purchased from MacFarlane Pheasants, Inc., Janesville, WI. Birds were housed in individual pens (1.2 m × 1.2 m × 1.2 m) at Iowa State University Poultry Farm and were kept on a 16-h lights on, 8 h lights off cycle. Prior to study inclusion, the pheasants were maintained on a basal commercial ration containing no anthelmintics, antibiotics or growth promoters. After a 1-week acclimatization period, all birds underwent a physical examination. Body weights ranged from 428 to 912 g, with males ranging from 715 to 912 g (average: 810 g, SD: 75 g) and females ranging from 428 to 688 g (average: 586 g, SD: 71 g). Animals were observed daily throughout the study for appearance and fecal score and had unrestricted access to feed and water throughout the study period. Study protocols were approved by the Institutional Animal Care and Use Committee of Iowa State University.

### 2.2 Randomization

**Figure 1:**
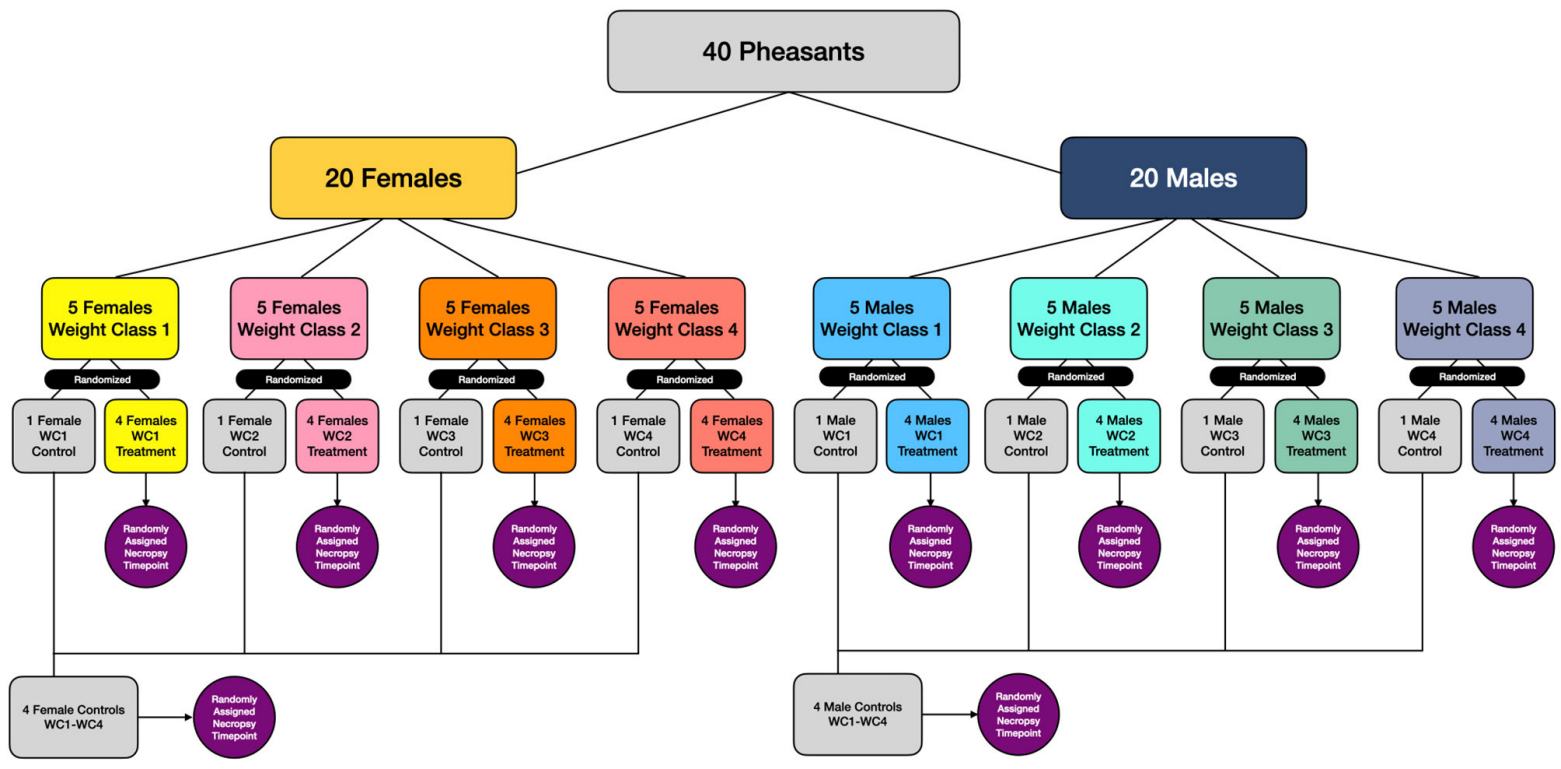
Randomization and treatment group assignment for pheasants administered fenbendazole medicated feed (100 ppm) for 7 days.

The birds were blocked by sex, ranked by weight within sex, and were subsequently blocked into 4 groups of 5 birds each with similar weights and same gender within the group–totaling 4 groups of females and 4 of males. The birds in each weight and sex group were randomly assigned to treatment groups in a 1:4 ratio, with one bird assigned to control (normal feed) and 4 birds assigned to fenbendazole (FBZ) medicated feed. One bird of each sex from each treatment weight group and one bird of each sex from the control groups were then randomly assigned a necropsy time–totaling 4 FBZ females, 4 FBZ males, 1 control female and 1 control male per time point (Figure 1).

### 2.3 Feed

Fenbendazole (Safeguard^®^, Merck Animal Health, Summit NJ) was supplied as a commercially available Type B medicated feed article containing 1.96% fenbendazole. The final FBZ treatment diets were formulated from a single lot of basal diet (Purina Game Bird Flight Conditioner, Purina Mills, St Louis MO) and mixed according to standard operating procedure with the appropriate amount of FBZ containing Type B medicated feed at a 1:10 ratio in a Hobart Model 11-600 mixer (Hobart, Troy, OH) to achieve a targeted concentration of 100 ppm. The FBZ treatment diet was fed ad-libitum to the FBZ treatment groups for 7 days. Birds assigned to the control group were exclusively fed the commercial game bird ration (Purina Game Bird Flight Conditioner) *ad libitum*. Diets for each treatment group were formulated in 3 batches sufficient to feed each group from day 0 to day 7. Samples were taken after mixing from each batch and assayed for FBZ levels, including the commercial game bird ration. FBZ concentration in the three medicated batches were 101.78, 102.05, 106.55 μg/g, and FBZ was not detected in all three of the basal diet batches.

### 2.4 Necropsy and sample collection

Following 7 days of the continuous feeding regimen, the medicated feed was removed from the treatment group. Within 6 h of feed removal, and then at intervals of 12, 24, and 48 h after medicated feed removal, one bird of each sex from each treatment weight group and one bird of each sex from the control groups—totaling 4 FBZ females, 4 FBZ males, 1 control female, and 1 control male per time point—were humanely euthanized by cervical dislocation. Following euthanasia, liver, pectoral muscle, and thigh muscle were removed and placed in individual containers. To ensure blinding of the analytical laboratory personnel, each sample was randomly assigned a code that did not reveal treatment group identity. The tissues were frozen at −80°C and shipped by overnight express to the analytical laboratory.

### 2.5 Tissue residue analysis

Liver, pectoral and thigh muscle was analyzed for FBZ, fenbendazole sulfoxide (FBZ-SO) and fenbendazole sulfone (FBZ-SO_2_) based on a previously described method (15). Briefly, homogenized tissue (1.0 g) was weighed into a centrifuge tube and extracted with 20 mL acetonitrile and 10 mL hexane. The hexane was discarded, and the acetonitrile volume adjusted to 25 mL. The sample was mixed with 15 g anhydrous sodium sulfate and a 10 mL aliquot of acetonitrile was evaporated to dryness by nitrogen evaporation (N-Evap, Organomation Associates, Inc., Berlin, MA). The dried extract was reconstituted in 0.2 mL N,N-dimethylformamide (DMFA) and 5 mL 3.5 mM phosphate buffer. The supernatant was passed through an Oasis^®^ HLB (Waters, Milford, MA) extraction cartridge and eluted with 5 mL acetonitrile. The eluate was evaporated to dryness, reconstituted with 50 μL dimethylsulfoxide, 2 mL ethyl acetate and 6 mL hexane, and passed through an Isolute^®^ CN (Biotage LLC, Charlotte, NC) extraction cartridge. Samples were eluted with 6 mL acidified methanol and again evaporated to dryness with nitrogen. Dried extracts were reconstituted with 0.2 mL DMFA and 0.8 mL mobile phase for High Performance Liquid Chromatography (HPLC) analysis.

Samples were injected on an Alliance^®^ HPLC (Waters, Milford, MA) with UV detection at 290 nm. Samples were eluted by gradient elution with mobile phase A being 12% acetonitrile in phosphoric acid solution [1.2 ml of phosphoric acid (85%) in water, pH adjusted to 3.0 with diethylamine and final dilution to 1 L] and mobile phase B being 80% acetonitrile in phosphoric acid solution. Beginning gradient conditions were 86% A and went to 100% B with a flow rate of 0.8 ml/min. The column was a Nova-Pak C18 (Waters, Milford, MA) 4 μm, 300 × 3.9 mm maintained at 24°C. Injection volume was 100 μl and the autosampler was kept at 10°C.

The analytical method was validated in accordance with the FDA CVM GFI #64 (VICH GL2) Validation of Analytical Procedures (16). The limit of quantification (LOQ) for FBZ, FBZ-SO, and FBZ-SO_2_ in the liver was 0.271, 0.027, and 0.126 μg/g respectively. For pectoral muscle the LOQ was 0.005, 0.007, and 0.011 μg/g and for thigh it was 0.013, 0.010, and 0.014 μg/g. Limits of detection (LOD) for FBZ, FBZ-SO, and FBZ-SO_2_ in the liver was 0.090, 0.010, and 0.040 μg/g respectively. For pectoral muscle the LOD was 0.002, 0.003, and 0.004 μg/g and for thigh muscle the LOD was 0.005, 0.005, and 0.005 μg/g. Recoveries ranged from 84.4 to 97.3%. Inter-assay precision as measured by percent of Relative Standard Deviation (RSD) ranged from 5.0–7.7% for thigh muscle, 2.4–7.3% for pectoral muscle and 4.3–8.0% for liver. Intra-assay precision ranges were 0.5–8.3% for thigh muscle, 1.7–9.4% for pectoral muscle and 0.5–9.8% for liver. Standard curves were linear from 0.012 μg/ml to 1.6 μg/ml (0.030 to 4.000 μg/g tissue equivalent) for FBZ, FBZ-SO, and FBZ-SO_2_. R^2^ values were 0.9972, 0.9983, and 0.9985 for FBZ, FBZ-SO, and FBZ-SO_2_ respectively. Tissue samples were analyzed within 1 week of harvesting except for two samples–pectoral muscle from bird 12 and thigh muscle from bird 5. These two samples required re-analysis which was performed 14- and 16-days post-euthanasia for birds 12 and 5, respectively.

### 2.6 Freezer stability

Freezer stability was assessed with both spiked and incurred samples since study samples were frozen prior to analysis. Samples for pectoral, thigh, and liver tissues from untreated pheasants were obtained prior to the start of the study. Briefly, 6 sets of 1 ± 0.05 g samples of pectoral, thigh, and liver tissue were spiked with FBZ, FBZ-SO_2_, and FBZ-SO each in replicates of 0.125, 0.5, and 2.0 μg/g. A set of one sample at each spiked level for each tissue was analyzed at the time of fortification, and the remaining samples were stored in a −80°C freezer. Subsequent sample sets were analyzed at 1, 3, 6, 9, and 12 weeks post-spiking. Moreover, Quality Control (QC) samples were prepared and analyzed with each sample set. Incurred samples were obtained from a separate target animal safety study (17). One set of each of the spiked and incurred samples (QCs-0.125, 0.5, and 2.0 μg/g and 1 × , 3 × , and 5 × incurred, all in duplicate) were analyzed at 0, 1, 3, 6, 9, and 12 weeks post-spiking.

### 2.7 Shipping stability

To assess shipping stability, four sets of untreated pheasant liver, pectoral muscle and thighs were spiked in duplicate at doses of 0.125, 0.5, and 2.0 μg/g each with FBZ, FBZ-SO_2_, and FBZ-SO. One set of samples was then analyzed for a time 0 concentration and the remaining sets sent to the in-life phase study director. The second and third set of the replicates were returned with the first shipment of tissue samples, and the fourth set was returned with the second shipment of tissue samples. Following the analysis of the tissue samples, the three sets of spiked samples were analyzed and compared to the time 0 set.

### 2.8 FDA and EMA assumption testing

Bartlett's test was performed to evaluate the homogeneity of variances of log_e_-transformed concentrations at each slaughter time point [Rstudio ver 1.4.1106 (18)]. Linear regression analysis was performed on the log_e_-transformed concentration vs. time plots that were created for each tissue matrix, and the regression line of each plot was visually inspected for linearity. Linearity of the regression line was also evaluated using ANOVA (lack-of-fit-test) performed in Rstudio [“ggplot2” and “dplyr” packages; ver 1.4.1106 (18)]. Plots of the ordered residuals vs. cumulative frequency distribution on a normal probability scale were evaluated to determine if there was a discrepancy between the log_e_ regression line and the log_e_ observed values [“Gvlma” package in Rstudio ver. 1.4.1106 (18)].

### 2.9 Statistical analysis for estimating a withdrawal interval recommendation

An estimated WDI for each tissue matrix was calculated for the FDA 95% confidence interval for the 99^th^ percentile population tolerance method (FDA 95/99) using an open-source statistical software program (package “Reschem”; R 3.3.1 program, University of Auckland, New Zealand) provided by FDA-CVM. Since no tolerance or marker residues currently exist for fenbendazole in pheasants in the USA, surrogate tolerances from other major poultry species were used in the calculation methods per European Food Safety Authority (EFSA) and FDA rulings (19, 20). For FBZ-SO_2_ (turkey and chicken maker residue) in the liver (turkey and chicken target tissue), both chicken and turkey liver tolerances (5.2 μg/g and 6 μg/g respectively), poultry liver MRL (0.5 μg/g) and pheasant liver LOD (0.04 μg/g) were used in the calculation method (Table 1). For pheasant thigh muscle data, since there is not an established FBZ or FBZ-SO_2_ tolerance for chicken or turkey muscle in the US, poultry muscle MRL (0.05 μg/g) and pheasant thigh muscle LOD (0.005 μg/g) were used for estimating WDIs. Similarly, for the pheasant pectoral muscle data, poultry muscle MRL (0.05 μg/g) and pheasant pectoral muscle LOD (0.004 μg/g) were used for estimating WDIs.

**Table 1:** Mean and range of fenbendazole sulfone (FBZ-SO_2_) in pheasant tissues (*n* = 8 birds per time point) following 7 days of 100 μg/g fenbendazole oral medicated feed.

**Tissue**	**Reference residue limits**	**Time post-removal of FBZ medicated feed (h)**	**Tissue FBZ-SO_2_ concentration (μg/g) Mean (range)**
Liver	FDA: 5.2 μg/g (Chicken) FDA: 6 μg/g (Turkey) EMA: 0.5 μg/g LOD: 0.04 μg/g	6	3.79 (1.64–5.43)
		12	2.65 (1.48–3.83)
		24	1.70 (0.97–2.36)
		48	0.49 (0.17–0.94)
Thigh	FDA: None listed EMA: 0.05 μg/g LOD: 0.005 μg/g	6	0.83 (0.33–1.25)
		12	0.71 (0.37–0.91)
		24	0.45 (0.21–0.67)
		48	0.11 (0.03–0.25)
Pectoral	FDA: None listed EMA: 0.05 μg/g LOD: 0.004 μg/g	6	0.80 (0.25–1.43)
		12	0.58 (0.36–0.76)
		24	0.36 (0.17–0.55)
		48	0.10 (0.05–0.21)

FDA, Food and Drug Administration; EMA, European Medicines Agency; LOD, limit of detection; EMA MRLs are for all species, and for the sum of all residues.

For the EMA MRL method, the data was analyzed using the program WT14 (Germany) with the same limits that were established for the FDA 95/99 method. WDIs were calculated using both the official EMA 95/95 method and an alternative EMA 95/99 method. The EMA 95/99 method, while not recommended by the EMA, was used for a direct comparison between the EMA and FDA methods for WDI calculation (21).

### 2.10 Estimating WDI using terminal elimination half-lives

Tissue residue concentration vs. time data was analyzed by non-compartmental pharmacokinetic analysis using the naïve pooled data approach to estimate the terminal elimination t_1/2_ for the marker FBZ-SO_2_ in each tissue [Phoenix^®^ WinNonlin^®^ version 8.3.4 (Certara USA, Inc., Princeton, NJ)]. To estimate a WDI using the terminal elimination half-life approach, the calculated t_1/2_ was multiplied by a factor of 10 to estimate WDIs FBZ-SO_2_ for each tissue.

## 3 Results

### 3.1 Clinical observations

There were no observed adverse effects that necessitated clinical intervention throughout the study period.

### 3.2 Freezer stability

Over the 12-week period, tissue samples from both spiked and incurred maintained consistent concentrations as indicated through comparison of the RSD values. Average RSD values for the marker residue FBZ-SO_2_ in QC freezer stability samples were <10% in liver tissue, <15% in pectoral muscle tissue, and <15% in thigh muscle tissue. For incurred samples, average RSD values for the marker residue FBZ-SO_2_ were <20%. The remainder of the freezer stability data for FBZ, FBZ-SO, and FBZ-SO_2_ can be found in Supplementary Table 1. While spiked samples were not tested week 1, results of all other timepoints in the freezer stability study indicate that there is no significant loss of drug substrate up to 12 weeks when stored at −80°C.

### 3.3 Shipping stability

Similar to the freezer stability data, results from the shipping stability data indicate that sample integrity was not affected by the shipping. RSD values were <20% for the marker residue FBZ-SO_2_ in liver, pectoral muscle, and thigh muscle tissue.

### 3.4 Tissue residue analysis

**Figure 2:**
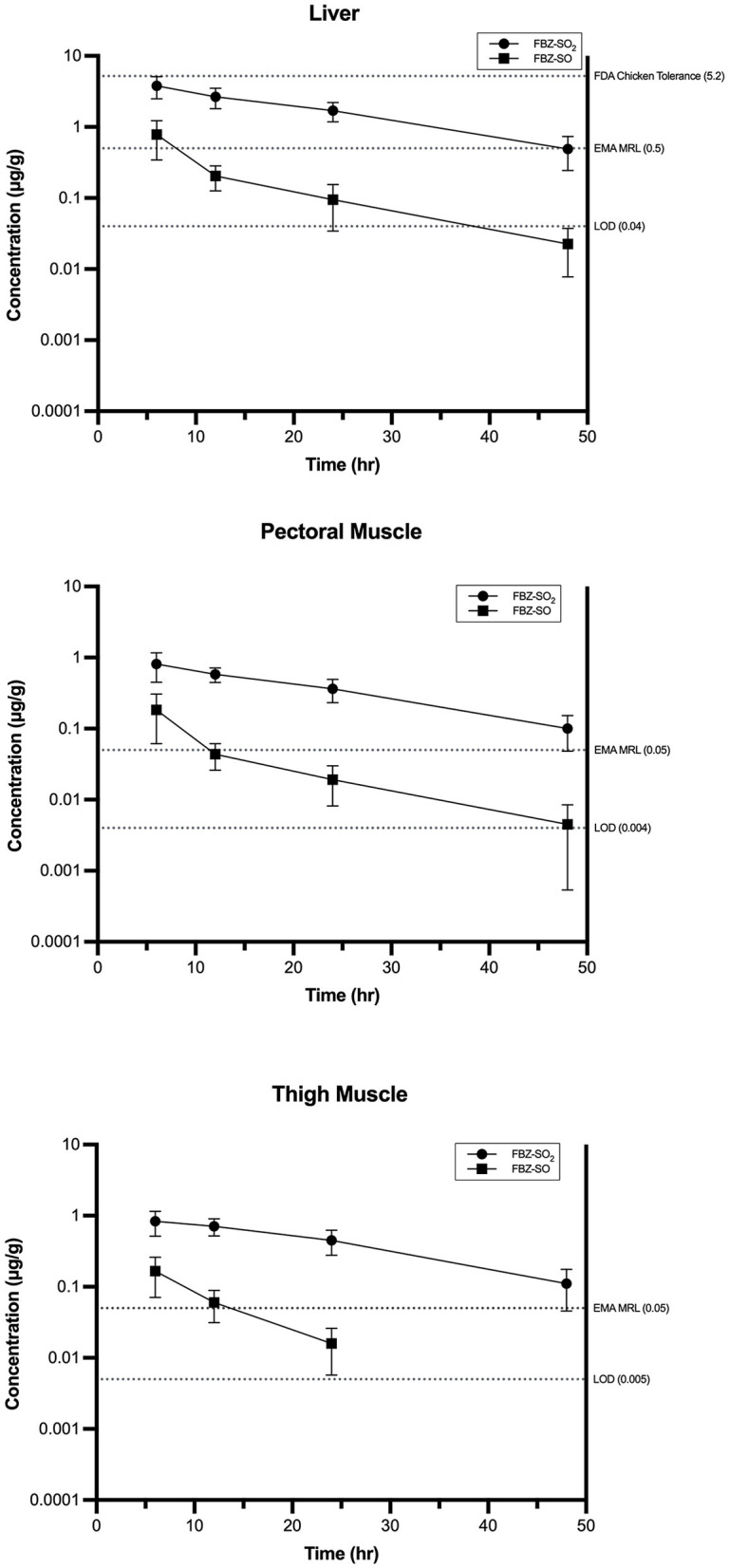
Fenbendazole sulfone (FBZ-SO_2_), and fenbendazole sulfoxide (FBZ-SO) residues in pheasant liver, thigh, and pectoral tissues following administration of 7 days of 100 μg/g fenbendazole oral medicated feed in comparison to tissue regulatory tolerances, maximum residue limits (MRLs), and the assay limit of detection (LOD).

The parent drug (FBZ) was below the LOD for all sampled tissues at all time points. The major metabolites FBZ-SO_2_ and FBZ-SO were detected in all tissues from treated birds up to 24 h after feed removal (Figure 2). At final sample timepoint (48 h following feed removal), FBZ-SO residues were below the LOD in thigh muscle samples but remained detectable in both pectoral muscle and liver samples (Figure 2). FBZ-SO_2_ residues were above the LOD in all tissue samples at all time points, with the highest concentrations of FBZ-SO_2_ detected in liver samples (Figure 2). Based on the tissue depletion data, FBZ-SO_2_ was determined to be the marker residue and the liver was determined to be the target tissue in pheasants.

Liver samples were below the FDA turkey liver tolerance of 6 μg/g at all time points (Table 1). Two liver samples were above the FDA chicken liver tolerance (5.2 μg/g) at 6 h post-feed removal, and the remainder of samples were below the FDA chicken liver tolerance (Table 1). Four of the birds on the control diet (Birds 41, 45, 4, and 10) were suspected of cross-contamination at slaughter or during tissue transport. Two of the birds on the control diet (Birds 41 and 45) had liver FBZ-SO residues above the LOD but below LOQ on analysis. Two of the birds on the control diet (Birds 4 and 10) had liver FBZ-SO residues at or above the LOQ on analysis, and had liver FBZ-SO_2_ residues above the LOD but below LOQ. Two of the birds on the control diet (Birds 41 and 4) had thigh muscle FBZ-SO residues above the LOD but below LOQ on analysis. As such, these birds were removed from any further analysis for study purposes. The remainder of tissues from control birds were below LOD for FBZ, FBZ-SO, and FBZ-SO_2_. Residue concentrations for control treated pheasants for FBZ, FBZ-SO, and FBZ-SO_2_ are reported in Supplementary Table 2.

Results of the tissue residue study are reported in Table 1. There were no significant differences in FBZ, FBZ-SO, or FBZ-SO_2_ residue concentrations between male and female pheasants in this study population. The mean terminal elimination half-life for FBZ-SO_2_ in tissues as estimated by non-compartmental analysis using the naïve pooled data approach was 14.5 h for liver, 13.2 h for thigh muscle, and 14.1 h for pectoral muscle. The remainder of tissue pharmacokinetic results are reported in Table 2. Mean concentrations of FBZ and its metabolites as detected in liver tissue samples were calculated and expressed as a percentage of the total (total = FBZ+FBZ-SO+FBZ-SO_2_) and are presented in Table 3.

**Table 2:** Predicted tissue pharmacokinetic parameters for fenbendazole sulfone (FBZ-SO_2_) estimated by non-compartmental analysis using a naïve pooled data approach from pheasants at slaughter (at 6, 12, 24, and 48 h) following 7 days of 100 ppm fenbendazole oral medicated feed administered to pheasants.

**Parameter**	**Liver**	**Pectoral muscle**	**Thigh muscle**
C_max_ (obs) μg/g	3.795	0.809	0.833
T_max_ (obs) (h)	6	6	6
λ_z_ (1/h)	0.048	0.049	0.052
Terminal Elimination Half-life (h)	14.54	14.07	13.21
AUC_0−∞_ (h^*^μg/g)	93.60	19.89	22.98
AUC_0−∞_ extrapolation (%)	11.2	10.3	9.53

C_max_, Maximum FBZ-SO_2_ concentration in tissue sample; T_max_, Time to maximum tissue concentration; AUC_0−∞_, Area under the tissue concentration vs. time curve extrapolated to infinity.

**Table 3:** Mean fenbendazole and fenbendazole metabolite concentrations as detected in liver samples from pheasants (*n* = 8 birds per time point) treated with fenbendazole medicated feed (100 μg/g) for 7 days.

**Time post-removal of FBZ medicated feed (h)**	**FBZ percent of total residues (mean ±sd)**	**FBZ-SO percent of total residues (mean ±sd)**	**FBZ-SO_2_ percent of total residues (mean ±sd)**
6 h	0.1% (0.006 ± 0.01 μg/g)	17.1% (0.78 ± 0.44 μg/g)	82.8% (3.79 ± 1.3 μg/g)
12 h	0.0% (0 ± 0 μg/g)	7.1% (0.205 ± 0.08 μg/g)	92.9% (2.65 ± 0.85 μg/g)
24 h	0.0% (0 ± 0 μg/g)	5.3% (0.09 ± 0.06 μg/g)	94.7% (1.70 ± 0.51 μg/g)
48 h	0.0% (0 ± 0 μg/g)	4.4% (0.02 ± 0.02 μg/g)	95.6% (0.49 ± 0.25 μg/g)

Concentrations are expressed as percentage of the total [total = fenbendazole (FBZ) + fenbendazole sulfoxide (FBZ-SO) + fenbendazole sulfone (FBZ-SO_2_)].

### 3.5 FDA and EMA assumption testing

The requirement of at least 5 or more animals in each group was met. Based on the Bartlett test, criteria for homoscedasticity of the dataset was met, with *p* = 0.47 for liver, *p* = 0.40 for thigh and *p* = 0.25 for pectoral samples. Visual inspection of the plots of the ordered residuals vs. cumulative frequency distribution on a normal probability scale revealed a straight line for liver, thigh, and pectoral muscle samples. Assumption of log-linearity was met based on the *p*-values for the lack-of-fit test, with *p* = 0.73 for liver, *p* = 0.37 for thigh muscle and *p* = 0.77 for pectoral muscle. Therefore, it was concluded that there was normality of errors, and all tissues met the FDA and EMA assumptions.

### 3.6 Statistical analysis: estimation of withdrawal interval

Calculation of WDI for FDA tolerance and EMA MRL methods was performed using FBZ-SO_2_ residue data from liver, thigh, and pectoral muscle samples of treated pheasants. The FDA 95/99 method for the FDA tolerance for FBZ-SO_2_ in chicken liver of 5.2 μg/g estimated the WDI as 24 h for pheasant liver (Table 4). Estimated ELDU WDIs using the terminal elimination half-life method, as well as the FDA tolerance and EMA MRL methods are presented in Table 4.

**Table 4:** Estimated withdrawal intervals for extra-label drug use (ELDU) of fenbendazole sulfone in pheasants administered fenbendazole medicated feed (100 μg/g) for 7 days.

**WDI estimation method [Confidence limit (%)/population percentile (%)]**	**Residue limit**	**Liver**	**Thigh muscle**	**Pectoral muscle**
FDA (95/99)	FDA Chicken Liver Tolerance: 5.2 μg/g	23.7 h (1 d)	NC	NC
	FDA Turkey Liver Tolerance: 6 μg/g	20.8 h (1 d)	NC	NC
	EMA All Species MRL: 0.5 μg/g (liver) 0.05 μg/g (muscle)	75.0 h (4 d)	98.0 h (5 d)	93.3 h (4 d)
	Analytical LOD (Pheasant): 0.04 μg/g (liver) 0.005 μg/g (thigh) 0.004 μg/g (pectoral)	135.0 h (6 d)	152.0 h (7 d)	153.0 h (7 d)
EMA (95/95)	FDA Chicken Liver Tolerance: 5.2 μg/g	16.9 h (1 d)	NC	NC
	FDA Turkey Liver Tolerance: 6 μg/g	14.2 h (1 d)	NC	NC
	EMA All Species MRL: 0.5 μg/g (liver) 0.05 μg/g (muscle)	68.1 h (3 d)	89.9 h (4 d)	85.9 h (4 d)
	Analytical LOD (Pheasant): 0.04 μg/g (liver) 0.005 μg/g (thigh) 0.004 μg/g (pectoral)	127.5 h (6 d)	143.4 h (6 d)	145.3 h (7 d)
EMA (95/99)	FDA Chicken Liver Tolerance: 5.2 μg/g	23.8 h (1 d)	NC	NC
	FDA Turkey Liver Tolerance: 6 μg/g	21.0 h (1 d)	NC	NC
	EMA All Species MRL: 0.5 μg/g (liver) 0.05 μg/g (muscle)	75.5 h (4 d)	98.1 h (5 d)	93.4 h (4 d)
	Analytical LOD (Pheasant): 0.04 μg/g (liver) 0.005 μg/g (thigh) 0.004 μg/g (pectoral)	134.8 h (6 d)	151.6 h (7 d)	152.7 h (7 d)
Terminal elimination half-life method	Theoretical 99% depletion (Pheasant)	145.4 h (7 d)	132.1 h (6 d)	140.7 h (6 d)

The calculated ELDU WDI is listed (h), then rounded up to the nearest 24 h interval to recommend a slaughter time. NC, Not calculated due to no tolerance listed for residues for the selected tissue matrix and withdrawal calculation method. FDA, US Food and Drug Administration; EMA MRL, European Medicines Agency Maximum Residue Limit; LOD, Limit of Detection; Terminal Elimination Half-Life Method is based on multiplying the target tissue half-life [14.54 h for liver, 13.21 h for thigh muscle, and 14.07 h for pectoral muscle] by a factor of 10.

## 4 Discussion

The results from this study support the use of FBZ-SO_2_ as the marker residue in the liver of pheasants following the extra-label administration of fenbendazole medicated feed (100 ug/g) for 7 days. This finding is consistent with the known pharmacokinetics of fenbendazole in chickens and turkeys, where fenbendazole undergoes rapid oxidation in the liver to FBZ-SO which subsequently undergoes further oxidation to the marker residue FBZ-SO_2_ (9). Consistent with other species and the extensive hepatic metabolism of FBZ, the liver had the longest terminal elimination half-life for the marker residue FBZ-SO_2_ of tissues tested for pheasants in this study (9, 22). Therefore, should a tolerance, MRL, or residue screening procedure be developed for fenbendazole in pheasants as part of a label claim for this species, the most appropriate marker residue and target tissue would be fenbendazole sulfone in the liver.

The longest estimated WDI in this study was 153 h (rounded to seven days) for the FBZ-SO_2_ metabolite using the pheasant LOD for pectoral muscle for the FDA 95/99 regulatory method. Since the pheasant pectoral muscle LOD (0.004 ppm) was the lowest limit of detection in our investigation, it resulted in the most conservative estimate of total tissue depletion regardless of regulatory calculation method (FDA 95/99, EMA 95/95, and EMA 95/99 calculation approaches). The similarity in the results between the FDA and EMA methods when the same residue limit and percentile population is used demonstrates harmonization in their mathematical approaches, and that variation in WDI calculations are primarily a result of the percentile of population used in the regulatory method and the region's tolerance/MRL.

A human food safety analysis that was previously performed using this dataset has demonstrated that, when the US turkey liver tolerance (6 ppm) is applied to pheasant tissues, an adult human would have to consume 442 grams of pheasant tissues per day to exceed the no-observed-adverse-effect level (NOAEL) for carcinogenicity (0.7 ppm) (23). However, when the LOD is applied as the tolerance limit, an adult would have to consume 63.9 kg of pheasant tissues daily to exceed the NOAEL for carcinogenicity (23). For context, in Belgium where pheasant meat consumption data is estimated for the population, the 95^th^ percentile for pheasant consumers have an estimated daily consumption of 119 grams/day (24), which is well below any the consumption level to exceed the lowest NOEL (0.7 ppm) even when the highest tissue tolerance (6 ppm, liver) is applied. This finding highlights the risks of using an extremely low analytical LODs as a determinant of food safety for ELDU in minor species in replacement of an adequate human food safety risk assessment. Using an analytical LOD as the determinant of food safety for ELDU in minor species may result in undue burden on producers to hold animals for a longer duration prior slaughter without reasonable risk of adverse effects in the human population.

In contrast, the least conservative approach for WDI estimation in this study was the EMA 95/95 method using the FDA turkey tolerance for liver, resulting in a 1-day (14.1 h) WDI. The short WDI resulting from this approach was likely due to a combination of using the highest tissue tolerance/MRL (6 ppm), and the minimization of the weight of extreme extrapolation from outliers in the calculation of WDIs using the 95^th^ percentile of the population. No liver tissue samples had a concentration of the marker residue FBZ-SO_2_ greater than the turkey liver tolerance (6 ppm) at any time point. Should a tolerance for FBZ be ever be established in pheasants at the same concentrations as in turkeys, a zero-day WDI would likely be appropriate according to the FDA tolerance method (25). By comparison, 2/8 liver tissue samples had FBZ-SO_2_ concentrations greater than the FDA chicken liver tolerance (5.2 ppm) at 6 h post- feed withdrawal, all samples were below the FDA chicken liver tolerance by 12 h post-feed withdrawal. Therefore, if chickens are deemed to be the most analogous species to pheasants by a regulatory authority, then a 1-day WDI would be most appropriate. However, it should be noted that while these calculations provide evidence that a short WDI may be acceptable for use of fenbendazole in pheasants at the study dose from a human food safety perspective, this approach is not currently recommended given the lack of approval for fenbendazole in this species. This is because this method may still result in violative residues from a regulatory perspective given the zero tolerance for residues in this species and sensitivity of the analytical methods used by the FDA and EMA.

The estimated WDIs for FBZ in pheasants with the half-life (t_1/2_) multiplication method were similar to those obtained using the regulatory methods with the LOD as the residue limit. There were minimal differences between tissue WDI estimates for the t_1/2_ multiplication method, with an estimated liver WDI of 145.4 (7 days), and the estimated thigh and pectoral muscle WDIs of 132.1 h (6 days) and 140.7 h (6 days) respectively. When compared to the regulatory methods, t_1/2_ multiplication method estimated a WDI of 7 days for liver compared to 6 days for the EMA 95/95, EMA 95/99 and FDA 95/99, and a WDI of 6 days for thigh and pectoral muscle compared to 7 days for both EMA 99% and FDA 99%. These small differences are a result of the selected population used in the calculation method, and the effects of rounding to the nearest day. The EMA and FDA methods use statistical approaches to represent a specific range of the population mean terminal elimination t_1/2_ to estimate depletion to a defined tolerance/MRL. The t_1/2_ multiplication method, however, uses the sample mean obtained from a small number of individual birds via a naïve pooled data approach to calculate the terminal elimination t_1/2_, which is then used to estimate its depletion to a theoretical 99% tissue residue depletion. Since the liver LOD is the highest of all the tissues (0.04 ppm), the use of the mathematical approach to deplete to theoretical zero results coupled with the longer tissue elimination t_1/2_ for liver results in a more conservative estimation for liver depletion than either of the statistical approaches. Conversely, the mathematical approach using the elimination t_1/2_ based on the sample mean resulted in a less conservative estimated WDI for thigh and pectoral muscle than the statistical methods, which is likely due to the differences in sample mean vs. population mean since the LOD for these tissues is very close to theoretical zero.

There were some limitations of this study. Two of the birds on the control diet (Birds 41 and 45) had liver FBZ-SO residues above the LOD but below LOQ. Two of the birds on the control diet (Birds 4 and 10) had liver FBZ-SO residues at or above the LOQ on analysis, and had liver FBZ-SO_2_ residues above the LOD but below LOQ. Two of the birds on the control diet (Birds 41 and 4) had thigh muscle FBZ-SO residues above the LOD but below LOQ on analysis. Contamination of the control diet was unlikely due to only 4/8 control birds having positive residues and that samples from the control diet tested after study completion had no fenbendazole detected. Given that the magnitude of the control positive sample values to the treated sample values and that the retention times in the chromatograms were in line with all the other samples in the runs, these residues were likely to have stemmed from contamination during slaughter or during initial tissue homogenization as part of sample preparation as was suspected. Birds 4 & 14 and birds 10 & 45 were slaughtered on the same date, and their tissues were also processed on the same date. Despite the care that was taken to clean the equipment between each sample, it is most likely that there may have been some cross-contamination during the slaughter or sample preparation process. This sample contamination was minimal but samples from these animals were eliminated and were not used. An additional limitation is that, since the pheasants in this study were all the same age, caution should be exercised when extrapolating this recommendation to large populations of different age groups and production classes. Finally, the use of a naïve pooled data approach for pharmacokinetic modeling meant that individual variability could not be accounted for in the pharmacokinetic parameter estimates, as pooled tissue drug concentration data for each timepoint was treated as if it originated from one animal. This approach was necessary because each tissue could only be sampled at a single timepoint for each individual bird—making it impossible to generate individual tissue concentration vs. time curves.

In conclusion, following ELDU administration of fenbendazole as an oral medicated feed at 100 μg/g for 7 days in 12-week-old pheasants, the most conservative estimated WDI across all calculation methods for all tissues was 7 days following removal of the medicated feed for the marker residue FBZ-SO_2_ in the pectoral muscle. Comparison of the FDA 95/99, EMA 95/95, and EMA 95/99 methods revealed harmonization in the WDI estimation for the marker residue of FBZ-SO_2_ in pheasant liver using the limit of detection for the analytical method as the residue limit. Future studies in larger groups of both sexes of pheasants from multiple age and reproductive classes are needed in order to extrapolate these recommendations to larger populations.

## Data availability statement

The raw data supporting the conclusions of this article will be made available by the authors, without undue reservation.

## Ethics statement

The animal study was approved by the IACUC log number is 1-11-7073-Q issued by the Iowa State University Office for Responsible Research. The title of the project was Human Food Safety (Tissue Residue) of Fenbendazole in Ring-necked Pheasants, approval date 2-25-2011. The study was conducted in accordance with the local legislation and institutional requirements.

## Author contributions

MC: Formal analysis, Methodology, Software, Writing – original draft, Writing – review & editing. MM: Conceptualization, Supervision, Visualization, Writing – original draft, Writing – review & editing. BM-L: Writing – original draft, Writing – review & editing. RG: Formal analysis, Writing – original draft, Writing – review & editing. SW: Formal analysis, Supervision, Writing – original draft, Writing – review & editing. LT: Methodology, Supervision, Writing – original draft, Writing – review & editing.
