# Supplementary material for: Estimation of withdrawal interval recommendations following administration of fenbendazole medicated feed to ring-necked pheasants (Phasianus colchicus)
Source: Front Vet Sci. 2024 Jul 31;11:1444009. doi: 10.3389/fvets.2024.1444009 (PMC11322809; doi:10.3389/fvets.2024.1444009)
Supplement: Supplementary file 1 [file Table_1.docx]

Supplemental Table 1: Freezer Stability Testing Summary Data for Fenbendazole in Pheasant Tissues for Fenbendazole (FBZ), Fenbendazole Sulfoxide (SO) and Fenbendazole Sulfone (SO2)
