# Supplementary material for: Estimation of withdrawal interval recommendations following administration of fenbendazole medicated feed to ring-necked pheasants (Phasianus colchicus)
Source: Front Vet Sci. 2024 Jul 31;11:1444009. doi: 10.3389/fvets.2024.1444009 (PMC11322809; doi:10.3389/fvets.2024.1444009)
Supplement: Supplementary file 2 [file Table_2.docx]

**Supplemental Table 2**: Fenbendazole (FBZ), Fenbendazole Sulfoxide (FBZ-SO), and Fenbendazole Sulfone (FBZ-SO_2_) Residues in Untreated Control Pheasants (n=8). FBZ-SO LOD: 0.01 ug/g (Liver) 0.005 ug/g (Thigh Muscle), FBZ-SO LOQ: 0.027 ug/g (Liver), 0.01 ug/g (Thigh muscle). FBZ-SO_2_ LOD: 0.04 ug/g (Liver), 0.005 ug/g (Thigh Muscle), FBZ-SO_2_ LOQ: 0.126 ug/g (Liver), 0.014 ug/g (Thigh Muscle).

| Control Bird Number | Tissue | FBZ (ug/g) | FBZ-SO  (ug/g) | FBZ-SO_2_  (ug/g) |
| --- | --- | --- | --- | --- |
| 16 | Liver | 0 | 0 | 0 |
| 16 | Thigh Muscle | 0 | 0 | 0 |
| 16 | Pectoral Muscle | 0 | 0 | 0 |
| 41 | Liver | 0 | 0 | 0 |
| 41 | Thigh Muscle | 0 | 0.02  (>LOD, <LOQ) | 0 |
| 41 | Pectoral Muscle | 0 | 0 | 0 |
| 4 | Liver | 0 | 0.027  (>LOD, =LOQ) | 0.053  (>LOD, <LOQ) |
| 4 | Thigh Muscle | 0 | 0.023  (>LOD, <LOQ) | 0 |
| 4 | Pectoral Muscle | 0 | 0 | 0 |
| 40 | Liver | 0 | 0 | 0 |
| 40 | Thigh Muscle | 0 | 0 | 0 |
| 40 | Pectoral Muscle | 0 | 0 | 0 |
| 10 | Liver | 0 | 0.031  (>LOD, >LOQ) | 0 |
| 10 | Thigh Muscle | 0 | 0 | 0 |
| 10 | Pectoral | 0 | 0 | 0 |
| 45 | Liver | 0 | 0.018  (>LOD, <LOQ) | 0.040  (>LOD, <LOQ) |
| 45 | Thigh Muscle | 0 | 0 | 0 |
| 45 | Pectoral Muscle | 0 | 0 | 0 |
| 7 | Liver | 0 | 0 | 0 |
| 7 | Thigh Muscle | 0 | 0 | 0 |
| 7 | Pectoral Muscle | 0 | 0 | 0 |
| 26 | Liver | 0 | 0 | 0 |
| 26 | Thigh Muscle | 0 | 0 | 0 |
| 26 | Pectoral Muscle | 0 | 0 | 0 |
